# Use of endoluminal vacuum therapy after anastomotic leak in a gynecologic oncology patient with rectosigmoid resection: A case report

**DOI:** 10.1016/j.gore.2022.101113

**Published:** 2022-12-06

**Authors:** Divya Gowthaman, Lisa R Gabor, Ken Y Lin, Julie Yang, Gary A Dellacerra, Sara S Isani, Dennis Y Kuo

**Affiliations:** a Department of Obstetrics and Gynecology and Women's Health, Division of Gynecologic Oncology, Albert Einstein College of Medicine, Montefiore Medical Center, 1300, Morris Park Avenue, USA; b Department of Medicine, Division of Gastroenterology, Albert Einstein College of Medicine, Montefiore Medical Center, 1300, Morris Park Avenue, USA; c Department of Radiology, Division of Abdominal Radiology, Albert Einstein College of Medicine, Montefiore Medical Center, 1300 Morris Park Avenue, USA

## Abstract

• Anastomotic leak is an infrequent complication after colon resection and is associated with high morbidity and mortality.
• Endoluminal vacuum therapy (EVAT) promotes wound closure by covering anastomotic leaks intraluminally and applying vacuum.
• EVAT has been shown to be safe with mild adverse events.
• EVAT should be considered in hemodynamically stable gynecologic oncology patients with a confined anastomotic leak.

## 1 Introduction:

Anastomotic leak is an infrequent complication after colon resection and is associated with high morbidity and mortality as well as poor long-term survival outcomes (Chadi et al., 2016). Anastomotic leak rates as high as 6.8 % have been reported in gynecologic oncology literature; risk factors include older age, low albumin, multiple bowel resections, distance from the anal verge (Frasson, Matteo MD, PhD*; Flor-Lorente, Blas PhD*; Ramos Rodríguez, José Luis PhD†; Granero-Castro, Pablo MD*; Hervás, David PhD‡; Alvarez Rico, Miguel Angel MD§; Brao, Maria Jesus Garcia MD¶; Sánchez González, Juan Manuel MD‖; Garcia-Granero, Eduardo MD, ePhD* the ANACO Study Group. Risk Factors for Anastomotic Leak After Colon Resection for Cancer: Multivariate Analysis and Nomogram From a Multicentric, Prospective, National Study With 3193 Patients. Annals of Surgery: August 2015). Leaks may lead to peritonitis, pelvic abscess, fistula, or sepsis. Anastomotic leaks are commonly managed with surgical intervention. However, in select cases, leaks can be managed non-surgically with antibiotics (Thomas and Margolin, 2016). Endoluminal vacuum therapy (EVAT) is a vacuum-assisted closure technique by covering an anastomotic leak with a sponge intraluminally and applying vacuum to promote wound closure. EVAT is a novel technique that has been increasingly employed in colorectal surgery for the management of confined anastomotic leaks and presents an opportunity to avoid operative intervention (Arezzo et al., 2010). Case series in colorectal anastomotic leaks utilizing this novel procedure report success rates greater than 70 % (Mussetto et al., 2017). (SeeTable 1.).

**Table 1:** Success and adverse events of EVAT in various anastomotic leak sites.

**Authors**	**Study Design**	**Site of Leak**	**Number of subjects**	**Success rate**	**Adverse events**
Laukoetter et al. (2017)	Retrospective	Esophageal	52	94.2 %	Hemorrhage, post -interventional strictures
Markus et al. (2022)	Prospective	Gastric	20	90.0 %	None
Weidenhagen et al. (2008)	Prospective	Colorectal	29	96.6 %	Rectovaginal fistula, reoperation, ischemic necrosis

The use of EVAT in gynecologic oncology patients is rare, with only one case series reporting its use in a patient with ovarian cancer as a prophylactic measure (Lehwald-Tywuschik et al., 2021). Here, we report a case of anastomotic leak after rectosigmoid colon resection treated with EVAT in a patient with high grade serous ovarian cancer who underwent tumor debulking. As seen in this case report, patient selection is critical in the application of this technique.

## 2 Case:

An 84-year-old woman with a past medical history of hypertension, type 2 diabetes, heart failure, and coronary artery disease with stent placement presented to the emergency room with left hip pain. Her associated symptoms included bloating, decreased appetite, and unexplained diarrhea 1–2 times per day for the last few months. She denied any abdominal pain or vaginal bleeding. An outpatient work-up included a PET/CT which demonstrated a 4.3 × 2.4 cm left adnexal mass with a bulky solid component of metabolically active tumor in addition to concern for local metastasis to the rectosigmoid colon. Her CA125 was 2446 U/ml. Her albumin level was 2.8 g/dL. The patient was then referred to gynecologic oncology for care. After obtaining cardiology clearance, she underwent a diagnostic laparoscopy followed by a laparotomy and tumor debulking. Given extensive involvement of the mass to the rectosigmoid colon, the patient underwent a total abdominal hysterectomy, bilateral salpingo-oophorectomy, omentectomy, pelvic and paraaortic lymph node dissection, and en bloc resection of the rectosigmoid colon with primary end-to-end reanastomosis. A GIA stapler was used to resect the proximal colon while the distal colon was resected with a TA stapler approximately 15 cm from the anal verge. After the proximal sigmoid colon was further mobilized, a purse string suture device and EEA stapler were used to complete a tension-free anastomosis.

Her immediate postoperative course was unremarkable with well controlled abdominal pain and passage of flatus. On post-operative day 6, however, she developed a fever and leukocytosis but exhibited stability in all of her hemodynamic parameters. Her abdominal examination was unchanged without evidence of peritonitis. CT of the abdomen and pelvis revealed a 6.7 × 3.3 × 3.4 cm anastomotic leak with air (Fig. 1a.), confined to the area of the anastomotic breakdown. Following guidance of consultation services, the patient was initially managed conservatively with bowel rest and broad-spectrum antibiotics. Total parenteral nutrition was started. Although the patient had return of bowel function and improved leukocytosis, repeat CT imaging on postoperative day 14 showed a persistent anastomotic leak (Fig. 1b). General surgery and gastroenterology teams were re-consulted and the decision was made to pursue EVAT therapy due to the confined nature of the leak and the patient’s overall clinical stability. Flexible sigmoidoscopy on postoperative day 15 confirmed an anastomotic leak characterized by a wide-mouthed 6 cm cavity at 15 cm from the anal verge (Fig. 2B). An endovacuum sponge was successfully placed (Fig. 2C) and connected to intermittent suction. EVAT exchanges were performed every 3–5 days for a total of 6 exchanges with improvement in cavity size and appearance noted on subsequent endoscopic evaluation (Fig. 2D). The type of anesthesia used for the exchanges was monitored anesthesia care (MAC), which the patient tolerated well. Perioperatively, prophylactic anticoagulation was continued while patient underwent EVAT exchanges. After closure of the defect, the patient was transitioned to a clear liquid diet on post-operative day 32, and antibiotics were discontinued. A rectal ulcer was observed on the last exchange (postoperative day 33), which was due to pressure from a previously dislodged sponge. The site of the anastomotic cavity appeared to be shallow, healing, and without necrotic tissue, therefore EVAT therapy was discontinued (Figure 25F). Repeat CT imaging with rectal contrast showed resolution of the collection at the level of the anastomosis, however small contrast extravasation was seen indicating a residual minimal leak (Fig. 1c), which was not concerning when discussed with the gastroenterology consult. The patient was advanced to a regular diet and antibiotics were discontinued. She was subsequently discharged from the hospital on post-operative day 40 to a subacute rehabilitation facility with plans for close outpatient follow up by gynecologic oncology and the gastroenterology team. The collection near the anastomosis was nearly resolved after EVAT therapy, and thus the patient did not have further monitoring with CT imaging for the purpose of evaluating the anastomotic leak.(SeeFig. 1d.).

**Fig. 1a:**
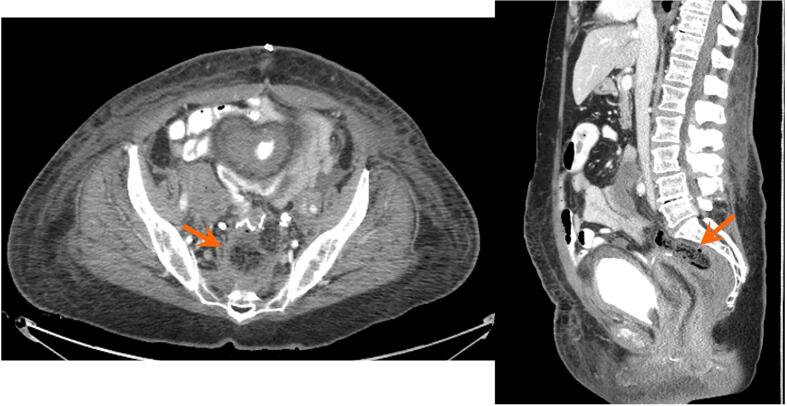
The first postoperative CT after colorectal anastomosis demonstrates a feculent-appearing collection posterior to the suture line (arrows), suspicious for anastomotic leak.

**Fig. 1b:**
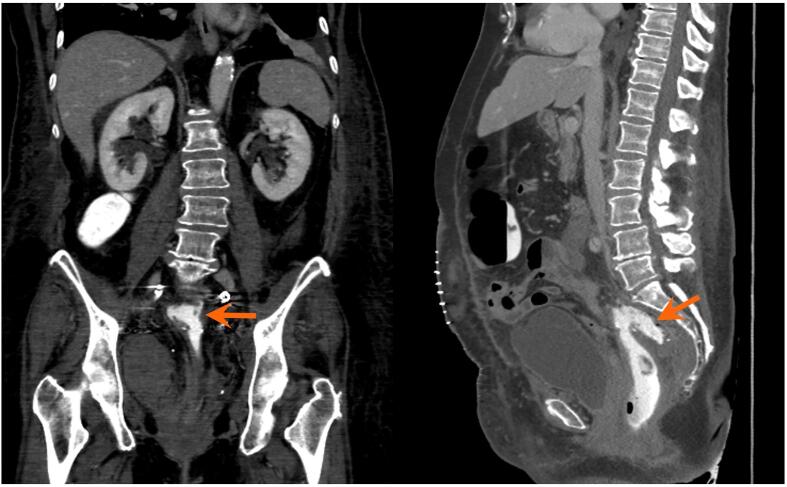
CT with rectal contrast performed a few days later demonstrates extravasation of contrast into this collection (arrows), confirming anastomotic leak.

**Fig. 1c:**
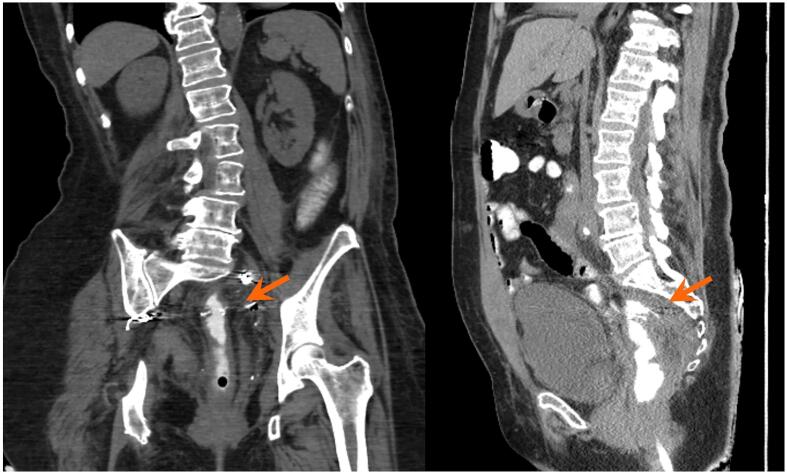
A few weeks after conservative management with an endoluminal vacuum assisted system, the contained collection is considerably smaller in size (arrows).

**Fig. 1d:**
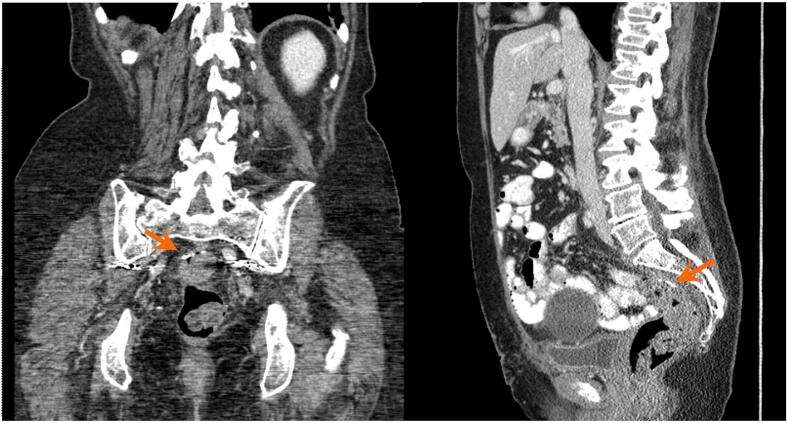
CT approximately 7 months after treatment shows complete resolution of the collection previously seen posterior to the suture line (arrows).

**Fig. 2:**
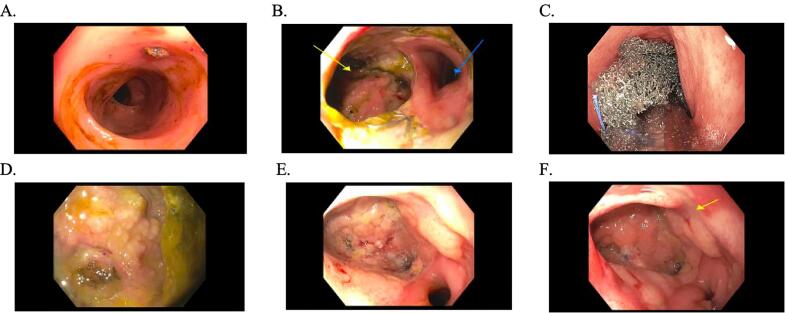
A. Normal sigmoid colon. B. Anastomosis with wide-mouthed 6 cm cavity on left with fistulous tracts. Sigmoid colon lumen on right. C. Successful placement of endovacuum. D. Fistulous area after the sponge was removed. E. Anastomosis at rectosigmoid. F. Cavity with healthy granulation tissue.

Her final pathology showed a stage IIIA1(i) high grade ovarian serous carcinoma. Given the patient’s improved clinical status, chemotherapy was started two weeks after discharge. The patient completed 6 cycles of systemic chemotherapy with paclitaxel and carboplatin. Her genetic test was negative; she did not have a BRCA mutation however was HR deficient. She was subsequently started on olaparib. She has had surveillance CT scans for monitoring of her disease status and has had no clinical evidence of disease.

## 3 Discussion:

Negative vacuum therapy for external wounds has been proven to be highly effective for promoting resolution of surgical wound separation. EVAT is an innovative approach that utilizes the same concept for internal use - either intracavitary or intraluminal. First introduced in 2003 in rectal surgery (Weidenhagen et al., 2004), this minimally invasive method is now used in esophageal, gastric, and rectal leaks. The therapy has been shown to have varying success rates, with the highest rate reported as 96.6 % in a study conducted by Weidenhagen et. al. Twenty-eight of the 29 patients in the study had definitive healing with a median duration of 34.4 days and 11.4 exchanges (Weidenhagen et al., 2008). The procedure has been shown to be safe with mild adverse events including ulcers (Mussetto et al., 2017).

Additionally, the efficacy of EVAT is improved when employed early after recognition of a leak, as seen in our patient whose endovacuum was placed on postoperative day 15. In a multicenter retrospective cohort study by Abdalla et. al, a 72.4 % success rate was noted when therapy was initiated within 15 days compared to 27.8 % when initiated more than 15 days after diagnosis (Abdalla, 2020). However, studies looking at long-term outcomes are limited. A multicenter study found that 25 % of patients were diagnosed with a pelvic abscess after a median follow up of 17 months (Riss et al., 2010).

Patient selection for EVAT is important, as EVAT is applicable for those with contained anastomotic leaks and for leaks accessible via endoscope (Weidenhagen et al., 2004). Patients must be hemodynamically stable with no clinical evidence of sepsis. Poor candidates for therapy include patients with enterocutaneous fistulas due to absence of negative pressure (Leeds et al., 2019). No current data exists regarding optimal albumin levels in ideal candidates for endovac therapy or how long to continue total parenteral nutrition (TPN) when started. The duration of TPN varies, however in studies of EVAT for upper gastrointestinal leakage, most patients received TPN for the entirety of EVAT (Reimer et al., 2022). Furthermore, EVAT has been shown to have limited efficacy in defects greater than 8 cm (Ooi et al., 2018) as the sponge may not be large enough to occlude the defect.

EVAT can lead to longer hospital stays due to the need for replacement of the endosponge every few days; our patient’s endosponge was exchanged every 3 to 5 days. It may also lead to a delay in the initiation of chemotherapy. The average duration of hospitalization after anastomotic leak is 40 days (Thornton et al., 2011 Mar). Although there are no studies directly comparing EVAT and other interventions such as drain placement or surgical intervention, EVAT has not been shown to reduce hospital stay. The total duration of EVAT in patients with anastomotic leak following anterior resection of the rectum was 34.4+/−19.4 days (Weidenhagen et al., 2008). In a study comparing percutaneous drainage and surgical repair of gastrointestinal anastomotic leaks, patients had longer average hospital stays for drainage compared to surgery (48 vs 32 days) (Burke, L.M.B., Bashir, M.R., Gardner, C.S. et al. Image-guided percutaneous drainage vs surgical repair of gastrointestinal anastomotic leaks: is there a difference in hospital course or hospitalization cost. Abdom Imaging 40, 2015). Although the length of hospital stay is comparable between these methods and endovac therapy can result in a delay to start of chemotherapy, the benefits of utilizing a continuous and successful minimally invasive method such as EVAT outweighs the disadvantages of delay in cancer treatment.

Additionally, endosponge dislodgement can occur, because the sponge is not secured in place as seen in our case. It is also recommended that patients be placed on parenteral hyperalimentation during the course of treatment. Nonetheless, patients with significant medical comorbidities who are hemodynamically stable, such as our patient, may benefit from a less invasive treatment such as EVAT as an alternative to surgery.

As discussed in case reports, EVAT has been primarily utilized after gastrointestinal and colorectal surgeries, however randomized clinical trials studying its efficacy are absent. Although this minimally invasive method has been incorporated into clinical practice for more than a decade, its use in gynecologic oncology is limited. This may be due to provider inexperience in using EVAT, a relatively lower incidence of anastomotic leaks in gynecologic oncology compared to colorectal surgery, and a limited number of optimal patients to apply this methodology. Despite the infrequent use, we feel that this treatment strategy should be considered and utilized in gynecologic oncology patients with a confined anastomotic leak and remain hemodynamically stable.

## 4 Conclusion

We present the case of a confined anastomotic leak after a rectosigmoid resection and anastomosis in a patient with ovarian cancer who was successfully treated with EVAT. EVAT may be an effective, nonsurgical treatment option for small, confined anastomotic leak after rectosigmoid resection in clinically stable patients. Further evaluation of this technique in gynecologic cancer patients with anastomotic leaks should be considered.

Consent:

An informed written consent was obtained from the patient for publication of this report and accompanying images.

Author Julie Yang, MD is a consultant of Cook Medical, ASCO, and Olympus Medical Systems Corp.

## CRediT authorship contribution statement

**Divya Gowthaman:** Writing – original draft. **Lisa R Gabor:** Writing – review & editing. **Ken Y Lin:** Visualization, Writing – review & editing. **Julie Yang:** Methodology, Writing – review & editing. **Gary A Dellacerra:** Methodology, Writing – review & editing. **Sara S Isani:** Supervision. **Dennis Y Kuo:** Conceptualization, Visualization, Writing – review & editing.

## Declaration of Competing Interest

The authors declare that they have no known competing financial interests or personal relationships that could have appeared to influence the work reported in this paper.
